# Remarks from the Editor-in-Chief

**DOI:** 10.1017/psy.2025.4

**Published:** 2025-03-24

**Authors:** Sandip Sinharay

**Affiliations:** ETS Research Institute

Dear *Psychometrika* Readers,

Welcome to the first *Psychometrika* issue of 2025. As promised in the last *Psychometrika* issue, *Psychometrika* is now an Open Access journal, published by Cambridge University Press. Consequently, we are now accepting all submissions of manuscripts (including revisions whose original versions were submitted to the old manuscript submission website) through the new website https://mc.manuscriptcentral.com/psychometrika.

The current issue includes 16 “Theory and Methods” section articles. In the first article, Weicong Lyu, Chun Wang, and Gongjun Xu perform fast detection of differential item functioning using multigroup regularized Gaussian variational estimation. The second article, written by Daniel McNeish and Tyler Matta, suggests a standardized root mean squared residual (SRMR) for latent variable models with covariates. In the third article, Ethan McCormick suggests several approaches for linear estimation with nonlinear inference. In the fourth article, Xue Wang, Jiwei Zhang, and Jing Lu suggest a novel variational Bayesian expectation maximization-maximization inference method for log-linear cognitive diagnostic models. The fifth article, by Kazuhiro Yamaguchi, Yanlong Liu, and Gongjun Xu, extends the loss function-based parameter estimation method for diagnostic classification models to consider prior knowledge and uncertainty of sampling. The sixth article, by Maarten Marsman, Don van den Berg, and Jonas Haslbeck, focuses on a Bayesian analysis of Markov random fields for ordinal data. In the seventh article, Fei Gu, Somboon Jarukasemthawee, Kullaya Pisitsungkagarn, and Ynte van Dam obtain the standard error estimates for rotated redundancy loadings that can facilitate the interpretation of the rotated redundancy variates. The eighth article, Letty Koopman, Bonne Zijlstra, and Andries van der Ark, includes an investigation of certain assumptions and properties of two-level nonparametric item response theory models. In the ninth article, Yang Ni and Su Chen perform casual structural modeling of survey questionnaires via a bootstrapped ordinal Bayesian network approach. In the 10th article, Ji Yeh Choi, Minjung Kyung, and Ju-Hyun Park generalize extended redundancy analysis to handle multiple mediators and mixed types of outcome variables under a Bayesian framework. Article 11, by Meta-Lina Spohn, Jeffrey Näf, Loris Michel, and Nicolai Meinshausen, discusses the development of a fully nonparametric, easy-to-use, and powerful test for the missing completely at random assumption on the missingness mechanism of a data set. In Article 12, Marie Beisemann, Heinz Holling, and Philipp Doebler introduce the multidimensional two-parameter Conway-Maxwell–Poisson model and develop marginal maximum likelihood estimation methods for the model. Article 13, by Yikai Lu, Jim Fowler, and Ying Cheng, introduces sequential item response theory models for multiple-choice, multiple-attempt items. In Article 14, Florian Schuberth, Tamara Schamberger, Ildikó Kemény, and Jörg Henseler introduce a new specification to structural equation modeling that allows for the direct modeling of sum scores. In Article 15, Jules Ellis, Andries van der Ark, and Klaas Sijtsma suggest an overall test of monotone homogeneity in item response theory. The issue ends with a correction, written by Albert Satorra and Peter M. Bentler, of their 2010 *Psychometrika* article.

Hope you enjoy the issue.

